# Oral Hyaluronic Acid Supplementation for the Treatment of Dry Eye Disease: A Pilot Study

**DOI:** 10.1155/2019/5491626

**Published:** 2019-09-25

**Authors:** Yeseul Kim, Chan Hee Moon, Bo-Yeon Kim, Sun Young Jang

**Affiliations:** ^1^Department of Ophthalmology, Soonchunhyang University Bucheon Hospital, Bucheon, Republic of Korea; ^2^Eunpyeong Hopeful Eye Clinic, Seoul, Republic of Korea; ^3^Division of Endocrinology and Metabolism, Department of Internal Medicine, Soonchunhyang University Bucheon Hospital, Bucheon, Republic of Korea

## Abstract

**Purpose:**

To evaluate the clinical efficacy of oral hyaluronic acid (HA) in patients with dry eye disease (DED).

**Study Design:**

Prospective randomized controlled trial.

**Methods:**

This trial enrolled 54 subjects and they were randomized into the study or control group. The inclusion criteria were as follows: (1) >18 years of age; (2) distance best-corrected visual acuity ≥ 20/40 Snellen equivalent in each eye; (3) IOP ≤ 21 mmHg in both eyes; (4) ocular surface disease index (OSDI) score of ≥18 and <65; (5) <10 seconds of tear break up time (TBUT); (6) >5 corneal spots of corneal fluorescein staining (CFS); and (7) ≤ 10 mm/5 min of the Schirmer test. All subjects were treated with a topical HA, and the study group was supplemented with oral HA. OSDI, TBUT, CFS, and the Schirmer test were evaluated for ocular surface parameters.

**Results:**

24 patients were assigned in the study group. Significant improvement of OSDI, TBUT, and CFS was observed at 1 month and 3 months after oral HA administration in the study group. At baseline and follow-up at 1 and 3 months, OSDI scores were 61.8 ± 16.2, 47.3 ± 11.6, and 42.3 ± 9.1, respectively (*P* < 0.001). TBUT was improved after treatment for 1 month and 3 months (4.2 ± 1.1; *P*=0.005 and 4.7 ± 1.1; *P* < 0.012). There were also statistically significant improvements in the CSF (1.8 ± 1.0, 0.8 ± 0.7; *P* < 0.001) at baseline compared with those at 1 month.

**Conclusions:**

A combined supplement of both oral and topical HA more efficiently improves corneal epithelial wound healing and related symptoms than topical HA alone, in DED.

## 1. Introduction

Dry eye disease (DED) is a common chronic ocular surface disease which is characterized by a loss of tear film homeostasis. It has multifactorial causes including tear film instability, hyperosmolarity, inflammation, and neurosensory abnormalities and results in ocular discomfort and visual impairment [1].

The treatment of DED is generally initiated with an instillation of lubricant eye drops or viscosity-enhancing agents [2]. Other major treatment options include anti-inflammatory therapy, tear conservation approach, and the treatment of lid abnormality. Dietary modification such as essential fatty acid, [3, 4] lactoferrin [5], and antioxidant supplement [6] is a minor treatment option; however, there is growing evidence that diet and nutritional supplementation play a role in DED.

Hyaluronic acid (HA) is a macromolecular mucopolysaccharide that is widely distributed throughout connective, epithelial, and neural tissues [7]. HA has been used in tear supplements to enhance lubrication as a topical agent, and it has been proven to promote corneal epithelial wound healing [8, 9]. However, HA can also be supplemented in oral route. Recently, HA has been taken orally to reduce joint pain in osteoarthritic knees and increase hydration for dry skin. Studies have proven that oral HA has anti-inflammatory [10] and skin moisturizing properties [11]. However, to the best of our knowledge, there is no trial of oral HA supplementation for the treatment of DED. In this study, we assumed that anti-inflammatory and moisturizing effects of oral HA also might work in DED. We investigated the effect of oral HA supplementation for the treatment of DED.

## 2. Materials and Methods

### 2.1. Subjects

This study was a single-center, nonblinded prospective randomized controlled trial. This study was approved by the Institutional Review Board of Soonchunhyang University Bucheon Hospital and conformed to the tenets of the Declaration of Helsinki. Informed consent was obtained from all patients and normal control subjects.

A total of 54 consecutive subjects were enrolled from the outpatient department from March 2016 to July 2016. The eyes of the subjects were randomized into the study or control group. The inclusion criteria for this study were as follows: (1) >18 years of age; (2) distance best-corrected visual acuity ≥20/40 Snellen equivalent in each eye; (3) IOP ≤ 21 mmHg in both eyes; (4) ocular surface disease index (OSDI) score of ≥18 and <65; (5) <10 seconds of tear break up time (TBUT); [12] (6) >5 corneal spots of corneal fluorescein staining (CFS); [13] (7) ≤10 mm/5 min of the Schirmer test. Patients with any other ocular diseases except DED or ocular injury were excluded from the study. Patients having ocular surgery or on topical lubricants during the previous 6 months were also excluded.

### 2.2. Topical Lubricant and Oral HA Supplementation

A topical lubricant of 0.15% HA ophthalmic suspension (New Hyaluni ophthalmic solution 0.15%, Taejoon, Ltd., Seoul, Korea) was allowed to both study and control group in each eye 5-6 times daily.

Oral HA 240 mg (Innerb, Suheung, Ltd., Seoul, Korea) once daily was supplemented in the study group for 3 months. It is reported that the physical properties and physiological activities of HA differ depending on its molecular weights. [14] In this study, 390 kDa of sodium hyaluronate was used.

### 2.3. Ocular Surface Parameters

Symptom assessment was performed using OSDI (Allergan, Irvine, Inc., Irvine, CA) [15], which evaluates symptoms of ocular discomfort, effects on visual function, and the impact of environmental triggers.

TBUT was measured using a fluorescein strip (fluorescein paper, Haag-Streit AG, Köniz, Switzerland). The fluorescein strip was moistened with one drop of saline and inserted into the lateral inferior fornix. The patient was asked to blink several times. Then the tear film was observed under the cobalt blue illumination of the slit lamp. The time between the last blink and the first dry spot on the cornea was taken to be the TBUT. The average TBUT of 3 repeated measurements was recorded for each eye.

Following the TBUT measurement, CFS was evaluated. Corneal staining was examined at the slit lamp using cobalt blue illumination and a Wratten 12 yellow barrier filter. The degree of corneal staining was assessed with the 5-point Oxford scale [16].

The Schirmer test was performed, without topical anesthesia, as a measure of basal tear production. A Schirmer strip (Colorbar, Inc., Eagle Vision, Memphis, TN) was folded from the edge and placed in the lateral third of the lower lid. The patients were instructed to close their eyes. The strip wetting was recorded after 5 minutes in millimeters, and a reading of less than 10 mm was considered abnormal.

### 2.4. Outcome Measures

OSDI, TBUT, CSF, and the Schirmer test (without anesthesia) were performed at baseline; then OSDI, TBUT, and CSF were undergone at 1 and 3 months after intervention. Primary outcomes were a mean change of OSDI, TBUT, and CSF between baseline and 1 month after treatment. Secondary outcomes were the mean change from 1 month to 3 months after treatment. Both eyes were evaluated, but only one eye with the worst score at the initial enrollment was included in the analyses.

### 2.5. Statistical Analysis

A Mann–Whitney *U* test was conducted to compare the measurements between the study and control group. The Wilcoxon signed-rank test was performed to compare changes in ocular surface parameters at baseline and 1 and 3 months after treatment.

SPSS 15.0 statistical software for Windows (SPSS Inc., Chicago, IL) was used for all statistical analyses. A two-sided test with a value of *P* < 0.05 was considered to be statistically significant.

## 3. Results

Twenty-four patients (6 males and 18 females) and thirty patients (8 males and 22 females) were assigned in the study and control groups, respectively. The mean patient age was 48.6 ± 13.0 in the study group and 53.29 ± 10.8 in the control group (*P*=0.073). The average of the Schirmer test, TBUT, CSF, and OSDI score at baseline are shown in Table 1. The parameters were not different significantly between the study and control group.

### 3.1. Primary Outcome

**Figure 1:**
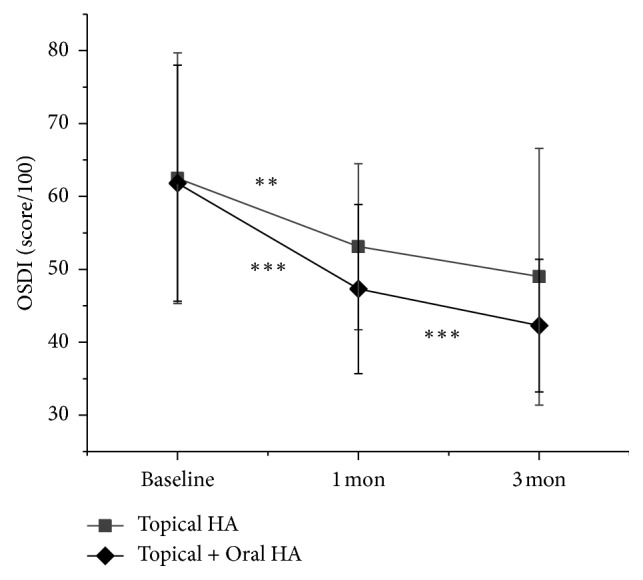
Change in ocular surface disease index (OSDI). Data are expressed as mean ± standard deviation (SD). Asterisks indicate values that are statistically significant between follow-up (^*∗∗*^*P* < 0.001 and ^*∗∗∗*^*P* < 0.001).

**Figure 2:**
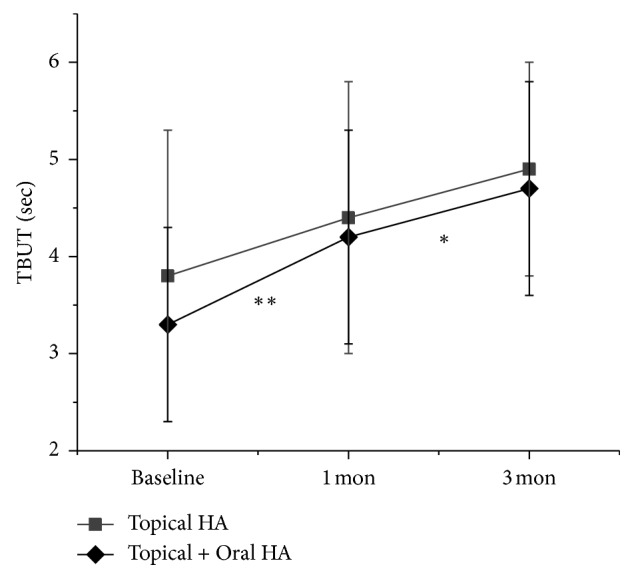
Change in tear break up time (TBUT). Data are expressed as mean ± standard deviation (SD). Asterisks indicate values that are statistically significant between follow-up (^*∗*^*P* < 0.05 and ^*∗∗∗*^*P* < 0.01).

**Figure 3:**
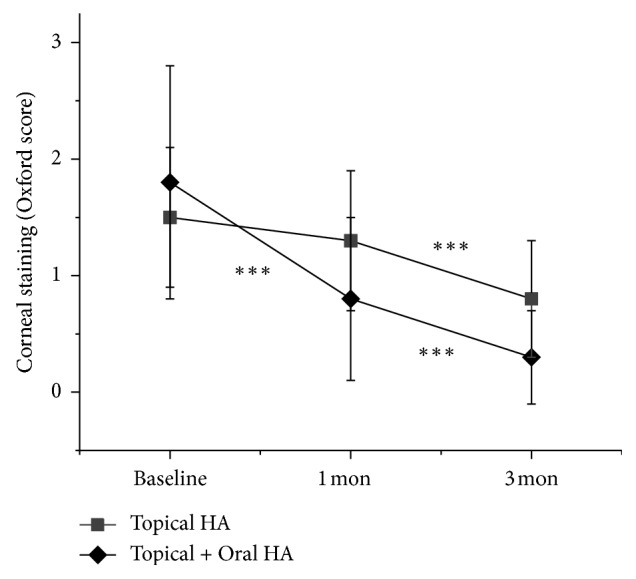
Change in corneal fluorescein staining (CFS) scores. Data are expressed as mean ± standard deviation (SD). Asterisks indicate values that are statistically significant between follow-up (^*∗∗∗*^*P* < 0.001).

One month after treatment, OSDI was significantly improved in both study group (1 month; 47.3 ± 11.6, *P* < 0.001) and control group (1 month; 53.1 ± 11.4, *P* < 0.007), (Figure 1). However, TBUT was significantly improved in the study group (1 month; 4.2 ± 1.1, *P*=0.005) but not in the control group (1 month; 4.4 ± 1.4, *P*=0.501), (Figure 2). CFS was also significantly improved in the study group (1 month; 0.8 ± 0.7, *P* < 0.001) but not in the control group (1 month; 1.3 ± 0.6, *P*=0.160) (Figure 3). The between-group difference in CSF at 1 month was significant (*P* < 0.001), but the differences in TBUT and OSDI were not significant (*P*=0.831 and *P*=0.135, respectively).

### 3.2. Secondary Outcome

Three months after treatment, OSDI was significantly improved when compared to one month after treatment in the study group (3 months; 42.3 ± 9.1, *P* < 0.001) but not in the control group (3 months; 49.0 ± 17.6, *P*=0.053) (Figure 1). TBUT was also significantly improved in the study group (3 months; 4.7 ± 1.1, *P* < 0.012) but not in the control group (3 months; 4.9 ± 1.1, *P* < 0.274) (Figure 2). However, CFS was also significantly improved in both study group (3 months; 0.3 ± 0.4, *P* < 0.001) and control group (3 months; 0.8 ± 0.5, *P* < 0.001) (Figure 3). The between-group differences in CFS and OSDI at 3 months were significant (*P* < 0.001 and *P*=0.046, respectively) but the difference in TBUT was not significant (*P*=0.166).

## 4. Discussion

In this study, we investigated the effect of oral HA supplementation for the treatment of DED. Combined oral and topical HA treated group showed significant improvement of CFS at 1 and 3 months. Otherwise, topical HA alone treated group showed significant improvement of CFS only at 3 months. The difference of CFS at 3 months between two groups was significant (*P* < 0.001, via the Mann–Whitney *U* test). In addition, OSDI was also significantly improved continually at 1 and 3 months after treatment in oral and topical HA supplemented group. However, topical HA alone treated group showed significant OSDI improvement at 1 month but not at 3 months. The difference of OSDI at 3 months between groups was also significant (*P*=0.046, via the Mann–Whitney *U* test). These results suggest that the combined supplement of both oral and topical HA more efficiently improves corneal epithelial wound healing and related symptoms than topical HA alone, in DED.

HA is a high molecular weight polysaccharide composed of repeating polymeric disaccharides of D-glucuronic acid and N-acetyl-D-glucosamine. Orally administered HA is degraded by intestinal bacteria and absorbed through the intestinal route. Though there are no digestive enzymes to degrade HA, hyaluronidase-producing bacteria including *Staphylococcus aureus* and *Clostridium perfringens* reside in the human intestine as a normal flora [17–19]. Balogh et al. [20] investigated the absorption of oral HA using radioactively labeled material in animal models and presented that approximately 90% of ingested HA was absorbed into and used by the body. In that study, they also showed that orally administered high molecular weight HA was also transferred into tissues without depolymerization through lymphatic uptake. Osami et al. [21] evaluated the effect of oral consumption of HA for dry skin using a skin surface analyser and reported that moisture content at a lower part of the eye significantly improved from 3 weeks to 6 weeks of ingestion compared to the placebo control group. This result implies that ingested HA was distributed at the ocular area.

Oral administration of high molecular weight HA modulates Th-1-associated inflammation [10]. The presence of CD4^+^ T cells at the ocular surface in DED and the improvement of surface inflammation with topical cyclosporine, which is a T-cell activity lowering agent, proposed a role for adaptive immunity in DED [22]. There is a growing body of evidence supporting the pathogenicity of CD4^+^ T cells in DED [23, 24]. Asari et al. [10] orally administered high molecular weight HA to MRL-lpr/lpr mice, a Th-1-type autoimmune disease model. In that study, cytokine array analysis showed the enhancement of interleukin-10 production and anti-inflammatory cytokine. DNA array analysis showed the upregulation of suppressor of cytokine signaling 3 (SOCS3) expression and the downregulation of pleiotrophin expression. These results suggest that the oral administration of high molecular HA may modulate Th-1-type autoimmune disease and inflammation.

HA promotes corneal epithelial wound healing. HA as well as its degradation products are capable of activating specific intracellular responses including epithelial cell proliferation, cell apoptosis, and neovascularization [25]. It is widely accepted that one of the key mediators for leading HA-associated cell activation is CD44. HA is a ligand for CD44 which is a multifunctional cell surface adhesion receptor. CD44 is ubiquitously expressed throughout the body [26] and also found on human corneas [27]. Studies have shown that CD44 upregulation associated with a proliferation of epithelial cells and migration [28, 29].

The present findings, however, should be interpreted taking into account the limitations of the study, particularly the small sample size and pilot characteristics of the trial. To ensure the results, further investigation with a larger sample size including double-blind placebo controlled group and experiments to clarify the underlying mechanisms are needed.

In conclusion, the concomitant supplement of both oral and topical HA more efficiently improves corneal epithelial wound healing and provides symptomatic relief than the topical administration of HA alone in DED.

## Figures and Tables

**Table 1:** Baseline demographics and clinical characteristics.

	Study group (topical + oral HA)	Control group (topical HA)	*P* value
Number of patients	24	30	
Age	48.6 ± 13.0	53.29 ± 10.8	0.073
Gender (% female)	75	76	0.755
Schirmer test (mm/5 min)	4.5 ± 1.0	4.9 ± 1.9	0.486
TBUT (sec)	3.3 ± 1.1	3.8 ± 1.5	0.104
CSF (Oxford score)	1.8 ± 1.0	1.5 ± 0.6	0.445
OSDI (score/100)	61.8 ± 16.2	62.5 ± 17.2	0.658

TBUT = tear break up time; CSF = corneal fluorescein staining; OSDI = ocular surface disease index. Data are expressed as mean ± standard deviation. *P* value was tested by the Mann–Whitney *U* test.

## Data Availability

Data supporting this research article are available from the corresponding author upon request.
